# The impact of common chronic conditions on health-related quality of life: a general population survey in Iran using EQ-5D-5L

**DOI:** 10.1186/s12962-021-00282-8

**Published:** 2021-05-13

**Authors:** Ali Akbari Sari, Fereshteh Karimi, Zahra Emrani, Hojjat Zeraati, Alireza Olyaeemanesh, Rajabali Daroudi

**Affiliations:** 1 Department of Health Management and Economics, School of Public Health, Tehran University of Medical Sciences, Poursina Ave, 1417613191 Tehran, Iran; 2 Department of Epidemiology and Biostatistics, Tehran University of Medical Sciences, Tehran, Iran; 3 National Institute for Health Research & Health Equity Research Centre, Tehran University of Medical Sciences, Tehran, Iran

**Keywords:** Health-related quality of life, Chronic disease, Economic evaluation, EQ-5D, Iran

## Abstract

**Background:**

Diseases have undeniable effects on Health-Related Quality of Life (HRQoL). Chronic diseases, in particular, limit the productive potentials and HRQoL of individuals. EQ-5D is a very popular generic instrument, which can be used to estimate HRQoL scores in any diseases. The current study investigates mean HRQoL scores in certain chronic diseases and examines the relationship between utility scores and chronic diseases in Iran.

**Method:**

This cross-sectional study was carried out among the general adult population of Tehran. 3060 individuals were chosen by a stratified probability sampling method. The EQ-5D-5L questionnaire was applied. The utility scores were estimated using the Iranian crosswalk-based value set. The effect of chronic diseases on the HRQoL scores was derived by the Ordinary Least Squares (OLS) method. Data was analyzed using Stata version 13 software.

**Results:**

The mean ± standard deviation utility and EQ-VAS scores were 0.85 ± 0.14 and 76.73 ± 16.55 in the participants without any chronic conditions. The scores were 0.69 ± 0.17 and 61.14 ± 20.61 in the participants with chronic conditions. The highest and lowest mean utility scores were related to thyroid disease (0.70) and Stroke (0.54), respectively. Common chronic conditions had significant negative effects on the HRQoL scores. Stroke (0.204) and cancer (0.177) caused the most reduction in the EQ-5D-5L utility scores. Lumbar disc hernia, digestive diseases, osteoarthritis, breathing problems, and anxiety/nerves cause 0.133, 0.109, 0.108, 0.087, and 0.078 reductions, respectively, in the EQ-5D-5L utility scores.

**Conclusion:**

This study provides insight into some common chronic conditions and their effects on the HRQoL. Policymakers and planners should pay attention to the effects of chronic conditions especially high prevalence one. They should adopt effective interventions to control this issue and increase health. The results of this study can also be beneficial in economic evaluation studies.

## Introduction

Chronic diseases are from the most common diseases in the world, which prevalence is estimated about 15–40% in the developed countries [1–3]. Some examples of chronic diseases are cardiovascular disease, muscular dysfunction, osteoporosis, renal failure, dementia, cancer and diabetes [4–6]. These diseases have significant negative effects on the Health-Related Quality of Life (HRQoL), as they would impose direct and indirect costs to the community [7–10]. In recent years, the therapeutic, economic, and social effects of chronic diseases have been highly considered by researchers and policymakers [11].

The main objective of health care is to improve the individual's quality and quantity of life. The quality of life is a multidimensional construct that includes social factors and the physical, mental, and functional dimensions [12]. It can be measured and developed in different ways, [13]. Health-related quality of life, briefly known as HRQoL, is an individual’s subjective view of the impact of the health condition on various aspects of his/her well-being and captures information about the impact of health status on “quality of life” [14]. In recent years, HRQoL has become an important health outcome indicator. There are several instruments for measuring HRQoL. Some are disease-specific (e.g. St George’s asthma quality of life scale, NEWQOL-6D, EORTC QOL-30), while some are generic (e.g., EQ-5D, SF-6D, WHOQOL) [15].

EQ-5D is the most widely used generic preference-based instrument developed by EuroQol in 1990 [16–19]. It is a multi-attribute instrument that considers five dimensions, including mobility, self-care, usual activities, pain/discomfort, and anxiety/depression. There are two versions of the EQ-5D instrument, which are EQ-5D-3L and EQ-5D-5L. The EQ-5D-5L questionnaire has five-level classifications of severity, including no problems, slight problems, moderate problems, severe problems, and extreme problems [20]. This questionnaire described 3125(5^5^) health states. For simplicity, health states are indicated by numerical symptoms. For example, 11,111 and 55,555 show the best and the worst health states, respectively. The former score represents a health state that the person has no problems, while the latter indicates a health state that the person has extreme problems in all dimensions. Other health states are between these two scores [21]. The social value sets for EQ-5D have been produced in many countries, including Iran [22, 23]. The instrument also includes a visual analogue scale (EQ-VAS), which provides a single global rating of self-perceived health. It is scored on a 0–100 mm scale representing “the worst” and “the best health you can imagine”, respectively. Respondents indicate where their current state of health lies relative to these anchors and therefore provide a direct valuation of the EQ-5D health states.

Health policymakers need to know which chronic conditions have the greatest impact on HRQoL, and identify where additional intervention may be required. Therefore, the aim of this study was to investigate the impact of a wide range of chronic conditions on HRQoL in the general Iranian population.

## Method

### Survey

This cross-sectional observational study was carried out from October 2015–March 2016 in Tehran, the capital of Iran, among the general adult population aged at least 18 years old. A sample of 3060 individuals was selected via a stratified probability sampling method. The target population was stratified by municipality, and from each stratum, a random sample with a size proportionate to the population was drawn. To select the respondents, each stratum (municipality region) was divided into several blocks. Then, based on the sample size from each stratum, the required number of blocks for data collection was randomly selected. In each block, ten households were randomly invited for the interview. Being aware of the age and gender distribution of the population, the reviewers were asked to select the respondent in each household in a way that this distribution can be observed in the sample, too. The households with non-responses were replaced with households in the replacement list. The interviews were conducted face-to-face by some trained interviewers.

### Questionnaire

The questionnaire consisted of three main parts. First, it collected information on demographic characteristics, such as gender, age, education, marital status, and employment status. The second part was regarding the general health status questions about the respondent’s viewpoints of his/her health and the presence of any illness or health problem in respondents. The presence of any illness or health problem was assessed with the question: “Do you have any illness, health problem, condition, or disability?”. The participants who had a disease or health problem were asked to choose the name of the disease from a list or simply mention the name of the disease. It was possible to choose or mention more than one option. The third part was the EQ-5D-5L questionnaire to determine the participants’ HRQoL scores.

### Statistical methods

To calculate the respondents’ HRQoL scores according to the EQ-5D-5L questionnaire, we used the five-level crosswalk-based value set derived from the EQ-5D-3L value set in Iran [23]. We applied the crosswalk methodology developed by Van Hout et al. [24] to the Iranian EQ-5D-3L value set developed via a face-to-face TTO method to obtain the Iranian crosswalk-based EQ-5D-5L value set [23].

The mean and standard deviation of the participants' HRQoL scores was calculated by common chronic diseases. Furthermore, the following regression model was estimated to determine the impact of common chronic diseases on participants’ HRQoL.

Q = αo + α_i_ Z_i_ + β_i_X_i_.

Q  = HRQoL score.

Zi = Common chronic diseases.

Xi = Demographic characteristics.

In this model, the common chronic diseases were included as independent variables. They were defined in forms of dummy variables. For example, a dummy variable was defined for diabetes, which took two values: “1” for patients with diabetes and “0” for patients without diabetes. The Iranian crosswalk-based value set scores were considered as dependent variables in the 1st model and the EQ-VAS scores in the 2nd model. These models were estimated using the OLS method. The Breusch-Pagan test was used to verify the Heteroscedasticity in the regression model. The data were analyzed using Stata13 software.

## Results

Table 1 shows the demographic characteristics and the mean HRQoL scores of the participants. About 51% of the participants were female; the mean ± standard deviation (SD) of the participants’ age was 44 ± 15.6 years; the average education years was 10.8 ± 4.8. About 77% of the subjects were married, 18% were single (never married), and 4.7% were divorced or widowed. The mean ± SD utility and EQ-VAS scores of the participants were 0.80 ± 0.17 and 71.73 ± 19.37.

**Table 1:** Demographic characteristics and mean HRQoL scores of participants (n = 3060)

Variable	N	%
Gender		
Male	1505	49.20
Female	1555	50.80
Employment status		
Employed	1079	35.30
Homemaker	1239	40.53
Retired	396	12.95
Unemployed	100	3.27
Student	221	7.23
Other	22	0.07
Marital status		
Never married	549	17.99
Married	2359	77.29
Widowed or divorced	144	4.72
Presence of any illness or health problem		
Yes	1115	36.46
No	1945	63.54
	mean	SD
Age (year)	43.9	15.6
Years of schooling	10.85	4.79
EQ-5D-5L utility scores	0.80	0.17
EQ-VAS scores	71.73	19.37

**Fig. 1:**
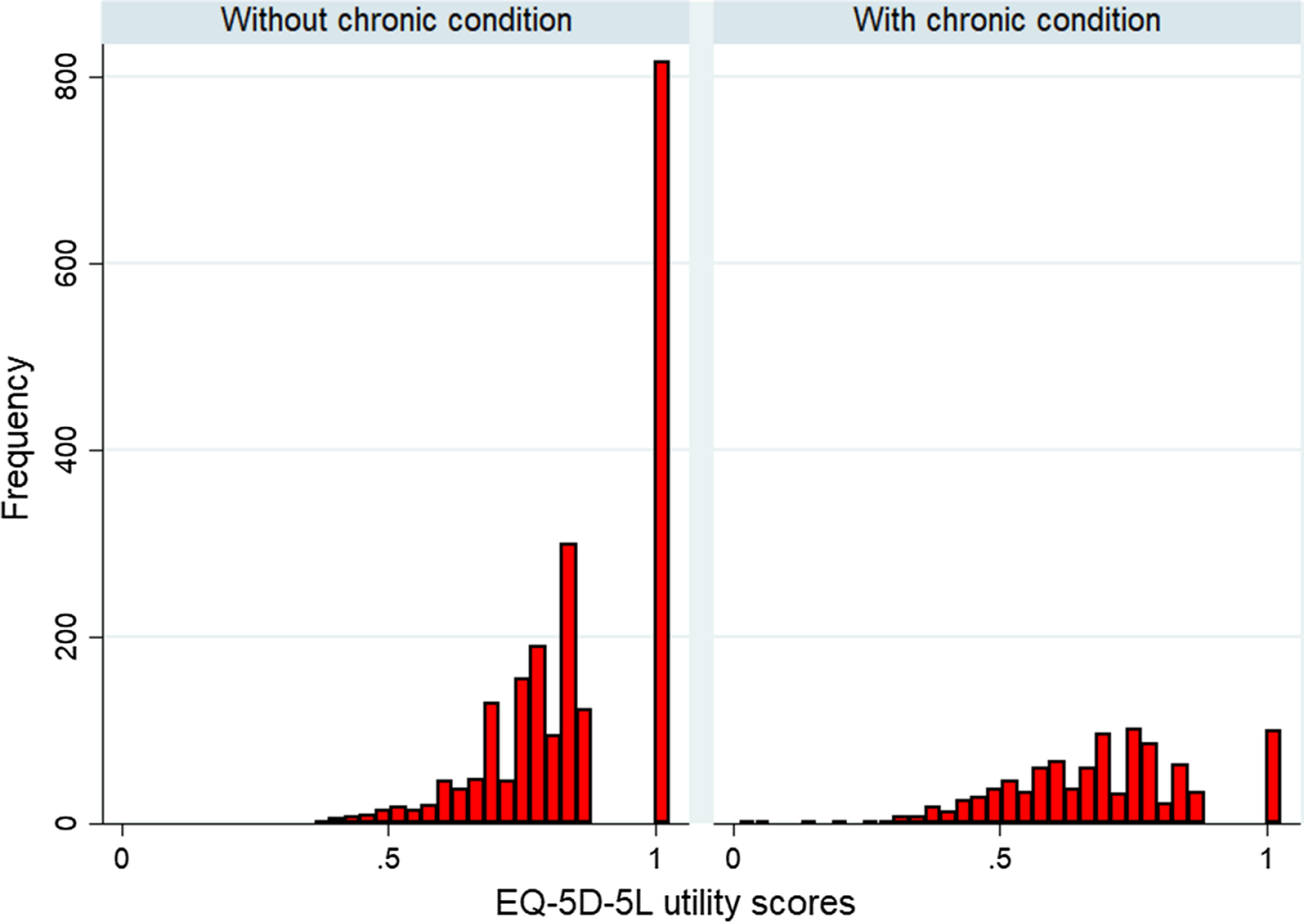
EQ-5D utility scores of the participants

**Fig. 2:**
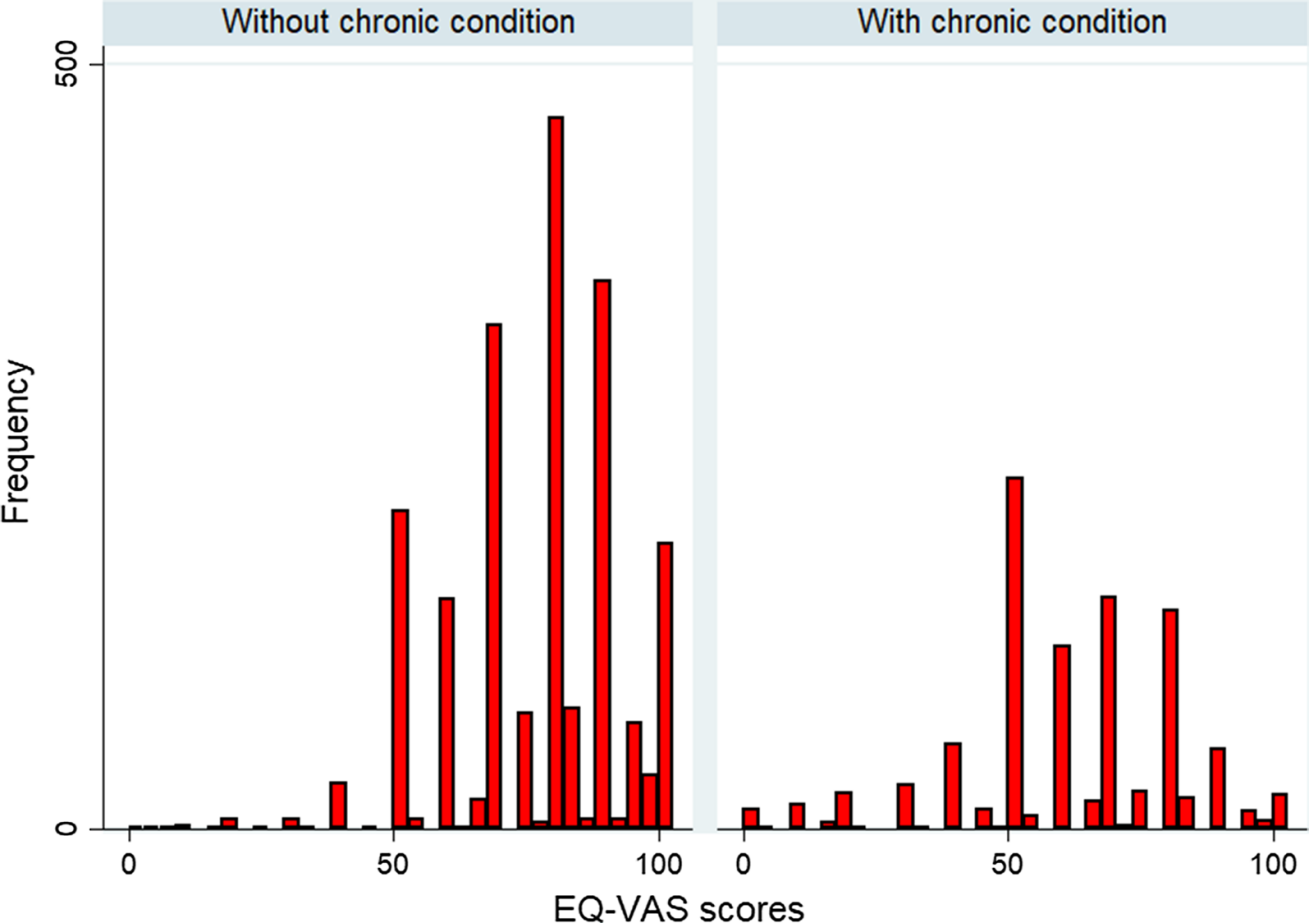
EQ-VAS scores of the participants

Figures 1 and 2 show the EQ-5D and EQ-VAS scores among participants without chronic condition compare to participants with chronic condition.

Table 2 shows the prevalence of chronic conditions among participants as well as the mean EQ-5D-5L utility and EQ-VAS scores by common chronic conditions. Approximately 32% of the participants had at least one chronic condition. The mean ± SD utility and EQ-VAS scores were 0.85 ± 0.14 and 76.73 ± 16.55 in the participants without any chronic conditions. The scores were 0.69 ± 0.17 and 61.14 ± 20.61 in the participants with chronic conditions. The most common conditions were psychological problems, including anxiety/nerves and depression (11.89%), osteoarthritis (7.22%), heart disease (6.40%), hypertension (6.8%), and diabetes (5.62%). The utility scores were the lowest in stroke (0.54) and cancer (0.58), while they were the highest in thyroid disease (0.70).

**Table 2:** Mean EQ-5D-5L utility and EQ-VAS scores by common chronic conditions

Chronic condition	N	% of total participants	EQ-5D-5L utility scores		EQ-VAS scores	
			Mean	SD	Mean	SD
Without any chronic condition	1945	63.54	0.86	0.14	77.50	16.14
With chronic condition	1115	36.46	0.69	0.17	61.61	20.39
Anxiety/nerves	231	7.55	0.66	0.17	59.57	22.34
Osteoarthritis	221	7.22	0.62	0.15	59.44	20.35
Heart disease	196	6.40	0.67	0.17	58.49	19.97
Hypertension	186	6.08	0.65	0.17	58.04	21.91
Diabetes	172	5.62	0.67	0.18	57.64	21.67
Insomnia	145	4.74	0.68	0.19	60.61	22.58
Depression	133	4.34	0.66	0.16	58.82	22.76
Breathing problems	60	1.96	0.67	0.19	60.03	19.81
Lumbar disc hernia	44	1.44	0.65	0.16	61.67	19.71
Digestive diseases	22	0.72	0.68	0.12	58.41	16.06
Cancer	19	0.62	0.58	0.25	50.32	30.07
Stroke	15	0.49	0.54	0.12	47.86	12.97
Thyroid disease	15	0.49	0.70	0.12	70.13	19.46
Other	117	3.82	0.67	0.18	58.69	21.73

Table 3 shows the impact of chronic conditions on the HRQoL. The estimated coefficients were negative and statistically significant for all conditions except for insomnia and Thyroid disease. Stroke and cancer cause the most reduction in the HRQoL scores, which shows 0.204 and 0.177 reductions in the EQ-5D-5L utility scores and 18.11 and 17.31 reductions in EQ-VAS scores, respectively. According to model 1, lumbar disc hernia, digestive diseases, osteoarthritis, breathing problems, and anxiety/nerves cause 0.133, 0.109, 0.108, 0.087, and 0.078 reductions, respectively, in the EQ-5D-5L utility scores. According to model 2, digestive diseases, other diseases, lumbar disc hernia, breathing problems, and diabetes cause 12.42, 10.01, 8.45, 7.80, and 7.55 reductions in the EQ-VAS scores.

**Table 3:** The results of linear regression estimation to determine the impact of chronic diseases on HRQoL

Independent variable	Dependent variable: quality of life score			
	Model 1: EQ-5D-5 L utility scores		Model 2: EQ_VAS scores	
	Coefficient	Robust SE	Coefficient	Robust SE
Gender (female)	− 0.024*	0.009	− 0.40	1.01
Age (year)	− 0.002*	0.000	− 0.16*	0.03
Years of schooling	0.003*	0.001	0.33*	0.08
Employment status				
Employed	Ref		Ref	
Student	− 0.007	0.010	1.25	1.30
Home maker	− 0.028*	0.010	− 1.92	1.17
Retired	0.015	0.010	3.04*	1.25
Unemployed	− 0.018	0.015	1.36	1.92
Others	− 0.091*	0.042	− 10.56	6.01
Marital status				
Never married	Ref		Ref	
Married	− 0.010	0.008	− 0.47	1.07
Divorce or widowed	− 0.046*	0.016	− 5.79*	2.11
Anxiety/nerves	− 0.078*	0.011	− 7.09*	1.53
Osteoarthritis	− 0.108*	0.010	− 5.68*	1.41
Heart disease	− 0.067*	0.012	− 7.20*	1.50
Hypertension	− 0.054*	0.012	− 4.79*	1.65
Diabetes	− 0.052*	0.014	− 7.55*	1.78
Insomnia	− 0.022	0.014	− 2.43	1.88
Depression	− 0.074*	0.013	− 6.78*	2.10
Breathing problems	− 0.087*	0.022	− 7.80*	2.49
Lumbar disc hernia	− 0.133*	0.025	− 8.45*	3.03
Digestive diseases	− 0.109*	0.029	− 12.42*	3.46
Cancer	− 0.177*	0.058	− 17.31*	7.17
Stroke	− 0.204*	0.036	− 18.11*	3.44
Thyroid disease	− 0.045	0.034	1.09	5.27
Other	− 0.097*	0.016	− 10.01*	1.97
Intercept	0.938*	0.018	80.29*	2.20
Number of observations	3041		3024	
Adjusted R2	0.307		0.199	
F statistic	57.35*		25.22*	

*Significant at P < 0.05

### Discussion

In this study, we investigated the effect of chronic conditions on the HRQoL scores. The mean ± SD utility and EQ-VAS scores were 0.85 ± 0.14 and 76.73 ± 16.55 in the participants without any chronic condition while the scores were 0.69 ± 0.17 and 61.14 ± 20.61 in the participants with chronic condition. The results showed that common chronic conditions had significant negative effects on the HRQoL scores. Stroke (0.204 ± 0.036), cancer (0.177 ± 0.58), lumbar disc hernia (0.133 ± 0.025), digestive diseases (0.109 ± 0.029), osteoarthritis (0.108 ± 0.010) caused the most reductions in the HRQoL scores. The mean HRQoL scores were the lowest among individuals with stroke (0.54 ± 0.12), cancer (0.58 ± 0.25), and osteoarthritis (0.62 ± 0.15) diseases.

Previous studies showed that common chronic diseases have major effects on the HRQoL, which are similar to ours [25–28]. However, due to the differences in individuals' preferences in different societies, the size of the effect of chronic diseases on the HRQoL may vary. A study in an elderly community-dwelling population in England showed the effects of common chronic diseases on the HRQoL using the EQ-5D questionnaire. The results suggested that most of these diseases reduce the HRQoL scores. Depression ( − 0.269, P < 0.001), neurological disease ( − 0.172, P < 0.0001), and osteoarthritis ( − 0.081, P = 0.0006) caused the greatest effects on the utility scores [26]. The results of a study on Sweden general population demonstrated that the HRQoL weighs were the lowest among individuals with depression, stroke, and low back pain. The scores were (0.38 ± 0.026), (0.44 ± 0.035), and (0.55 ± 0.011) in people with these diseases. Regression analysis showed that depression, low back pain, and stroke caused (0.4305 ± 0.0270), (0.2810 ± 0.0105), and (0.2743 ± 0.0366) reductions in the HRQoL (P < 0.0001) [25]. Another study on the general population in Finland showed that Parkinson's disease, anxiety disorders, arthrosis of the hip and knee, and depressive disorders were the most disabling chronic conditions based on EQ-5D, causing (0.201 ± 0.063), (0.169 ± 0.019), (0.155 ± 0.010), and (0.139 ± 0.016) reductions in mean utility scores, respectively. The mean utility scores were the lowest among individuals with Parkinson’s disease (0.440 ± 0.068), heart failure (0.585 ± 0.017), and stroke (0.587 ± 0.023) [29].

A study in Hong Kong using the EQ-5D-5L questionnaire included four chronic diseases as an independent variable, whose effects were statistically significant on the HRQoL. The mean utility score for heart disease, hypertension, diabetes, and cancer was 0.88, 0.88, 0.87, and 0.87, respectively. In our study, these scores were 0.67, 0.65, 0.67, and 0.58, according to the Iranian crosswalk-based value set. The figures indicated that these diseases induced greater loss in Iranian HRQoL scores than the Chinese scores [30].

The present study, for the first time, measured the association between chronic conditions and HRQoL via the EQ-5D-5L questionnaire in Iran. The EQ-5D-5L questionnaire is a general, reliable, and convenient measurement tool applied in the surveys of different diseases [30].

The main limitation of this study was the fact that we measured the prevalence of chronic diseases based on self-reporting. Although studies have shown the high degree of agreement between the actual prevalence of chronic diseases and people’s self-declaration [31], this method is not completely accurate because some people might not be aware of their illness or its name. In this study, we only examined the net effect of each of the chronic conditions on health-related quality of life. Given that the number of chronic conditions was high, we did not examine the interaction between them. However, there may be an interaction between some diseases, such as diabetes and heart disease, and their combined effect could be more or less than the sum of their net effects. Another limitation relates to the method of calculating the utility score. It is better to use the specific value set of each questionnaire to extract the utility scores. Because the EQ-5D-5L value set is not still available for Iranian population, we used the crosswalk method.

## Conclusion

This study examined the effect of chronic conditions on the HRQoL scores using the EQ-5D-5L questionnaire in Iran. Almost all chronic conditions included in this study had a negative effect on HRQoL. Policymakers need to consider the increasing prevalence of chronic diseases in Iran due to the aging population and lifestyle changes. Additionally, they should pay attention to the elderly people who suffer from several chronic diseases at the same time. Hence, it is worthy of identifying the diseases with the greatest effect on the HRQoL. The effective interventions can be adopted and better prioritized. The results of this study can also be beneficial for researchers because the estimated utility weighs can be used in economic evaluation studies.

## Data Availability

The datasets used and/or analyzed during the current study are available from the corresponding author on reasonable request.

## Abbreviations

HRQoL: Health-related quality of life
OLS: Ordinary least squares
